# 2-Amino-5-methyl­pyridinium 2-hydr­oxy-3,5-dinitro­benzoate

**DOI:** 10.1107/S1600536810014480

**Published:** 2010-04-28

**Authors:** Madhukar Hemamalini, Hoong-Kun Fun

**Affiliations:** a X-ray Crystallography Unit, School of Physics, Universiti Sains Malaysia, 11800 USM, Penang, Malaysia

## Abstract

In the title mol­ecular salt, C_6_H_9_N_2_^+^·C_7_H_3_N_2_O_7_^−^, the 2-amino-5-methyl­pyridinium cation is essentially planar, with a maximum deviation of 0.023 (1) Å. There is an intra­molecular O—H⋯O hydrogen bond in the 3,5-dinitro­salicylate anion, which generates an *S*(6) ring motif. In the crystal, the protonated N atom and the 2-amino group are hydrogen bonded to the carboxyl­ate O atoms *via* a pair of N—H⋯O hydrogen bonds, forming an *R*_2_^2^(8) ring motif. Weak inter­molecular C—H⋯O inter­actions help to further stabilize the crystal structure.

## Related literature

For background to the chemistry of substituted pyridines, see: Pozharski *et al.* (1997 ▶); Katritzky *et al.* (1996 ▶); Nahringbauer & Kvick (1977 ▶). For 3,5-dinitro­salicylic acid, see: Hindawey *et al.* (1980 ▶); Issa *et al.* (1981 ▶). For details of hydrogen bonding, see: Jeffrey & Saenger (1991 ▶); Jeffrey (1997 ▶); Scheiner (1997 ▶). For hydrogen-bond motifs, see: Bernstein *et al.* (1995 ▶). For bond-length data, see: Allen *et al.* (1987 ▶). For the stability of the temperature controller used in the data collection, see: Cosier & Glazer (1986 ▶).
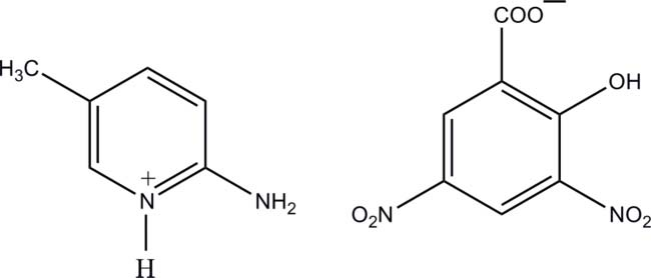


## Experimental

### 

#### Crystal data

C_6_H_9_N_2_^+^·C_7_H_3_N_2_O_7_^−^
*M* *_r_* = 336.27
Triclinic, 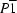

*a* = 5.8673 (7) Å
*b* = 8.0991 (9) Å
*c* = 15.2437 (17) Å
α = 86.844 (3)°
β = 84.252 (3)°
γ = 81.209 (3)°
*V* = 711.69 (14) Å^3^
*Z* = 2
Mo *K*α radiation
μ = 0.13 mm^−1^
*T* = 100 K
0.29 × 0.14 × 0.08 mm

#### Data collection

Bruker APEX DUO CCD area-detector diffractometer
Absorption correction: multi-scan (*SADABS*; Bruker, 2009 ▶) *T*_min_ = 0.963, *T*_max_ = 0.990
12709 measured reflections
4947 independent reflections
3922 reflections with *I* > 2σ(*I*)
*R*_int_ = 0.023

#### Refinement

*R*[*F* ^2^ > 2σ(*F* ^2^)] = 0.045
*wR*(*F* ^2^) = 0.150
*S* = 1.08
4947 reflections
219 parameters
H-atom parameters constrained
Δρ_max_ = 0.51 e Å^−3^
Δρ_min_ = −0.46 e Å^−3^

### 

Data collection: *APEX2* (Bruker, 2009 ▶); cell refinement: *SAINT* (Bruker, 2009 ▶); data reduction: *SAINT*; program(s) used to solve structure: *SHELXTL* (Sheldrick, 2008 ▶); program(s) used to refine structure: *SHELXTL*; molecular graphics: *SHELXTL*; software used to prepare material for publication: *SHELXTL* and *PLATON* (Spek, 2009 ▶).

## Supplementary Material

Crystal structure: contains datablocks global, I. DOI: 10.1107/S1600536810014480/hb5405sup1.cif

Structure factors: contains datablocks I. DOI: 10.1107/S1600536810014480/hb5405Isup2.hkl

Additional supplementary materials:  crystallographic information; 3D view; checkCIF report

## Figures and Tables

**Table 1:** Hydrogen-bond geometry (Å, °)

*D*—H⋯*A*	*D*—H	H⋯*A*	*D*⋯*A*	*D*—H⋯*A*
O1—H1*A*⋯O7	0.82	1.66	2.4200 (13)	152
N1—H1⋯O6^i^	0.86	1.82	2.6768 (14)	174
N2—H2*A*⋯O7^i^	0.86	2.11	2.9668 (15)	176
N2—H2*B*⋯O1^ii^	0.86	2.16	2.8468 (15)	137
N2—H2*B*⋯O2^ii^	0.86	2.40	3.1723 (16)	149
C2—H2⋯O4^iii^	0.93	2.49	3.4073 (16)	169
C4—H4⋯O3^iv^	0.93	2.39	3.2371 (16)	151
C5—H5⋯O2^ii^	0.93	2.44	3.2328 (17)	143

Symmetry codes: (i) 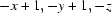; (ii) 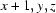; (iii) 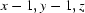; (iv) 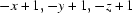.

**Fig. 1.**
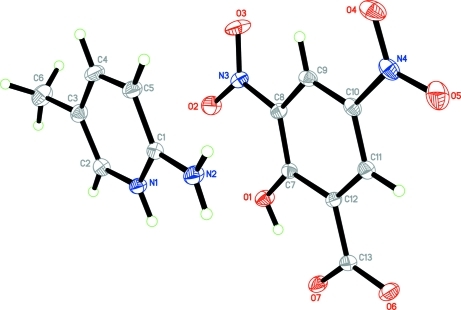
The asymmetric unit of (I). Displacement ellipsoids are drawn at the 50% probability level.

**Fig. 2.**
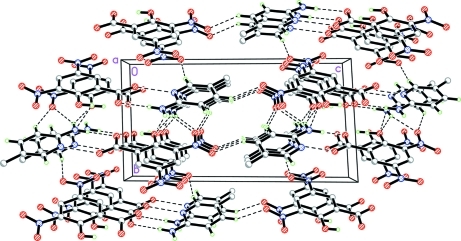
The crystal packing of (I), showing hydrogen-bonded (dashed lines) networks. H atoms are not involing the hydrogen bond interactions are omitted for clarity.

## supplementary crystallographic information

### Comment

Pyridine and its derivatives play an important role in heterocyclic chemistry (Pozharski *et al.*, 1997; Katritzky *et al.*, 1996). They are often involved in hydrogen-bond interactions (Jeffrey & Saenger, 1991; Jeffrey, 1997; Scheiner, 1997).  The nitro-substituted aromatic acid 3,5-dinitrosalicylic acid (DNSA) has proven potential for formation of proton-transfer compounds, particularly because of its acid strength (pKa = 2.18), its interactive ortho-related phenolic substituent group together with the nitro substituents which have potential for both π···π interactions as well as hydrogen-bonding interactions.  A large number of both neutral and proton-transfer compounds of Lewis bases with DNSA, together with their IR spectra have been reported (Hindawey *et al.*, 1980; Issa *et al.*, 1981) in the literature. Since our aim is to study some  interesting hydrogen bonding interactions, the crystal structure of the title compound is presented here.

The asymmetric unit (Fig. 1) contains one 2-amino-5-methylpyridinium cation and one 3,5-dinitrosalicylate anion. The proton transfer from the carboxyl group to atom N1 of 2-amino-5-methylpyridine resulted in the widening of C1—N1—C2 angle of the pyridinium ring to  123.48 (10)°, compared to the corresponding angle of 117.4° in neutral 2-amino-5-methylpyridine (Nahringbauer & Kvick, 1977). The 2-amino-5-methylpyridinium cation is essentially planar, with a maximum deviation of 0.023 (1) Å for atom N1. The bond lengths and angles are normal (Allen *et al.*, 1987). The phenol oxygen atoms are bent slightly away from the mean plane of the benzene ring [torsion angle O1-C7-C8-C9 = 179.70 (12)°].

In the crystal packing (Fig. 2), the protonated N1 atom and the 2-amino group (N2) is hydrogen-bonded to the carboxylate oxygen atoms (O6 and O7) via a pair of intermolecular N1—H1···O6 and N2—H2A···O7 hydrogen bonds forming a ring motif *R*_2_^2^(8) (Bernstein *et al.*, 1995).  There is an intramolecular O—H···O hydrogen bond in the 3,5-dinitrosalicylate anion, which generates an *S*(6) ring motif.  The crystal structure is further stabilized by weak C—H···O (Table 1) hydrogen bonds.

### Experimental

A hot methanol solution (20 ml) of 2-amino-5-methylpyridine (27 mg, Aldrich) and 3,5-dinitrosalicylic acid (58 mg, Merck) were mixed and warmed over a heating magnetic stirrer for a few minutes. The resulting solution was allowed to cool slowly at room temperature and yellow blocks of (I) appeared after a few days.

### Refinement

All hydrogen atoms were positioned geometrically [C–H = 0.93 or 0.96 Å, N–H = 0.86 Å and O–H = 0.82 Å] and were refined using a riding model, with U_iso_(H) = 1.2 U_eq_(C, N) or 1.5 U_eq_(O). The methyl H atoms were positioned geometrically [C–H = 0.96 Å] and were refined using a riding model, with U_iso_(H) = 1.5U_eq_(C). A rotating group model was used for the methyl group.

### Crystal data

**Table:**

C_6_H_9_N_2_^+^·C_7_H_3_N_2_O_7_^−^	*Z* = 2
*M**_r_* = 336.27	*F*(000) = 348
Triclinic, *P*1	*D*_x_ = 1.569 Mg m^−^^3^
Hall symbol: -P 1	Mo *K*α radiation, λ = 0.71073 Å
*a* = 5.8673 (7) Å	Cell parameters from 5139 reflections
*b* = 8.0991 (9) Å	θ = 2.7–32.4°
*c* = 15.2437 (17) Å	µ = 0.13 mm^−^^1^
α = 86.844 (3)°	*T* = 100 K
β = 84.252 (3)°	Block, yellow
γ = 81.209 (3)°	0.29 × 0.14 × 0.08 mm
*V* = 711.69 (14) Å^3^	

### Data collection

**Table:**

Bruker APEX DUO CCD area-detector diffractometer	4947 independent reflections
Radiation source: fine-focus sealed tube	3922 reflections with *I* > 2σ(*I*)
graphite	*R*_int_ = 0.023
φ and ω scans	θ_max_ = 32.5°, θ_min_ = 1.3°
Absorption correction: multi-scan (*SADABS*; Bruker, 2009)	*h* = −8→8
*T*_min_ = 0.963, *T*_max_ = 0.990	*k* = −12→11
12709 measured reflections	*l* = −22→23

### Refinement

**Table:**

Refinement on *F*^2^	Primary atom site location: structure-invariant direct methods
Least-squares matrix: full	Secondary atom site location: difference Fourier map
*R*[*F*^2^ > 2σ(*F*^2^)] = 0.045	Hydrogen site location: inferred from neighbouring sites
*wR*(*F*^2^) = 0.150	H-atom parameters constrained
*S* = 1.08	*w* = 1/[σ^2^(*F*_o_^2^) + (0.0741*P*)^2^ + 0.3055*P*] where *P* = (*F*_o_^2^ + 2*F*_c_^2^)/3
4947 reflections	(Δ/σ)_max_ < 0.001
219 parameters	Δρ_max_ = 0.51 e Å^−^^3^
0 restraints	Δρ_min_ = −0.46 e Å^−^^3^

### Special details

**Table:**

Experimental. The crystal was placed in the cold stream of an Oxford Cryosystems Cobra open-flow nitrogen cryostat (Cosier & Glazer, 1986) operating at 100.0 (1) K.
Geometry. All s.u.'s (except the s.u. in the dihedral angle between two l.s. planes) are estimated using the full covariance matrix. The cell s.u.'s are taken into account individually in the estimation of s.u.'s in distances, angles and torsion angles; correlations between s.u.'s in cell parameters are only used when they are defined by crystal symmetry. An approximate (isotropic) treatment of cell s.u.'s is used for estimating s.u.'s involving l.s. planes.
Refinement. Refinement of F^2^ against ALL reflections. The weighted R-factor wR and goodness of fit S are based on F^2^, conventional R-factors R are based on F, with F set to zero for negative F^2^. The threshold expression of F^2^ > 2σ(F^2^) is used only for calculating R-factors(gt) etc. and is not relevant to the choice of reflections for refinement. R-factors based on F^2^ are statistically about twice as large as those based on F, and R- factors based on ALL data will be even larger.

### Fractional atomic coordinates and isotropic or equivalent isotropic displacement parameters (Å^2^)

**Table:**

	*x*	*y*	*z*	*U*_iso_*/*U*_eq_	
N1	0.50349 (18)	0.28625 (13)	0.24313 (7)	0.0151 (2)	
H1	0.4877	0.2768	0.1882	0.018*	
N2	0.82364 (19)	0.41676 (14)	0.19955 (7)	0.0176 (2)	
H2A	0.8060	0.4015	0.1454	0.021*	
H2B	0.9352	0.4662	0.2121	0.021*	
C1	0.6777 (2)	0.36374 (15)	0.26413 (8)	0.0146 (2)	
C2	0.3509 (2)	0.22205 (16)	0.30452 (8)	0.0162 (2)	
H2	0.2353	0.1692	0.2859	0.019*	
C3	0.3654 (2)	0.23421 (16)	0.39271 (8)	0.0177 (2)	
C4	0.5424 (2)	0.31915 (17)	0.41660 (8)	0.0196 (2)	
H4	0.5553	0.3318	0.4761	0.023*	
C5	0.6949 (2)	0.38321 (16)	0.35500 (8)	0.0180 (2)	
H5	0.8088	0.4391	0.3726	0.022*	
C6	0.2014 (2)	0.1628 (2)	0.46135 (9)	0.0252 (3)	
H6A	0.0930	0.1115	0.4329	0.038*	
H6B	0.1192	0.2508	0.4971	0.038*	
H6C	0.2873	0.0806	0.4980	0.038*	
O1	0.17251 (16)	0.61810 (13)	0.14298 (6)	0.01924 (19)	
H1A	0.1638	0.6119	0.0899	0.029*	
O2	0.10528 (18)	0.60967 (13)	0.31694 (7)	0.0243 (2)	
O3	0.28454 (18)	0.76459 (15)	0.38862 (6)	0.0270 (2)	
O4	0.93341 (17)	0.99542 (13)	0.26211 (7)	0.0237 (2)	
O5	1.00019 (18)	1.02494 (14)	0.11971 (8)	0.0274 (2)	
O6	0.55546 (18)	0.76372 (13)	−0.07527 (6)	0.0222 (2)	
O7	0.26539 (17)	0.63243 (12)	−0.01529 (6)	0.01923 (19)	
N3	0.25086 (18)	0.70325 (14)	0.31952 (7)	0.0166 (2)	
N4	0.89119 (18)	0.97295 (13)	0.18590 (8)	0.0181 (2)	
C7	0.3389 (2)	0.70035 (15)	0.15497 (8)	0.0136 (2)	
C8	0.3891 (2)	0.74498 (15)	0.23904 (8)	0.0141 (2)	
C9	0.5696 (2)	0.83248 (15)	0.24908 (8)	0.0152 (2)	
H9	0.6004	0.8588	0.3049	0.018*	
C10	0.7029 (2)	0.87992 (15)	0.17497 (8)	0.0148 (2)	
C11	0.6613 (2)	0.84379 (15)	0.09005 (8)	0.0154 (2)	
H11	0.7517	0.8788	0.0409	0.018*	
C12	0.4831 (2)	0.75506 (14)	0.08054 (7)	0.0136 (2)	
C13	0.4365 (2)	0.71642 (15)	−0.01015 (8)	0.0160 (2)	

### Atomic displacement parameters (Å^2^)

**Table:**

	*U*^11^	*U*^22^	*U*^33^	*U*^12^	*U*^13^	*U*^23^
N1	0.0161 (4)	0.0167 (5)	0.0128 (4)	−0.0032 (4)	−0.0022 (3)	−0.0010 (3)
N2	0.0184 (5)	0.0215 (5)	0.0143 (4)	−0.0086 (4)	−0.0004 (3)	−0.0001 (4)
C1	0.0161 (5)	0.0138 (5)	0.0138 (5)	−0.0018 (4)	−0.0014 (4)	−0.0003 (4)
C2	0.0144 (5)	0.0164 (5)	0.0180 (5)	−0.0032 (4)	−0.0014 (4)	−0.0009 (4)
C3	0.0183 (5)	0.0184 (5)	0.0156 (5)	−0.0022 (4)	0.0005 (4)	0.0009 (4)
C4	0.0238 (6)	0.0234 (6)	0.0123 (5)	−0.0048 (5)	−0.0033 (4)	−0.0007 (4)
C5	0.0200 (5)	0.0205 (6)	0.0149 (5)	−0.0058 (5)	−0.0039 (4)	−0.0005 (4)
C6	0.0225 (6)	0.0321 (7)	0.0206 (6)	−0.0078 (5)	0.0030 (5)	0.0045 (5)
O1	0.0192 (4)	0.0257 (5)	0.0154 (4)	−0.0105 (4)	−0.0028 (3)	−0.0014 (3)
O2	0.0271 (5)	0.0276 (5)	0.0202 (5)	−0.0141 (4)	0.0037 (4)	−0.0011 (4)
O3	0.0285 (5)	0.0422 (6)	0.0122 (4)	−0.0098 (5)	−0.0019 (4)	−0.0062 (4)
O4	0.0210 (4)	0.0230 (5)	0.0296 (5)	−0.0032 (4)	−0.0110 (4)	−0.0068 (4)
O5	0.0235 (5)	0.0269 (5)	0.0337 (6)	−0.0121 (4)	−0.0010 (4)	0.0021 (4)
O6	0.0286 (5)	0.0266 (5)	0.0127 (4)	−0.0099 (4)	0.0000 (3)	−0.0007 (3)
O7	0.0215 (4)	0.0242 (5)	0.0142 (4)	−0.0094 (4)	−0.0031 (3)	−0.0008 (3)
N3	0.0163 (4)	0.0197 (5)	0.0134 (4)	−0.0017 (4)	−0.0011 (3)	−0.0005 (4)
N4	0.0144 (4)	0.0149 (5)	0.0261 (5)	−0.0027 (4)	−0.0048 (4)	−0.0025 (4)
C7	0.0133 (5)	0.0139 (5)	0.0135 (5)	−0.0018 (4)	−0.0015 (4)	−0.0009 (4)
C8	0.0137 (5)	0.0158 (5)	0.0128 (5)	−0.0021 (4)	−0.0006 (4)	−0.0009 (4)
C9	0.0145 (5)	0.0151 (5)	0.0162 (5)	−0.0007 (4)	−0.0038 (4)	−0.0030 (4)
C10	0.0125 (5)	0.0132 (5)	0.0195 (5)	−0.0031 (4)	−0.0028 (4)	−0.0017 (4)
C11	0.0148 (5)	0.0140 (5)	0.0171 (5)	−0.0019 (4)	−0.0007 (4)	−0.0010 (4)
C12	0.0147 (5)	0.0144 (5)	0.0119 (5)	−0.0029 (4)	−0.0014 (4)	−0.0008 (4)
C13	0.0187 (5)	0.0162 (5)	0.0136 (5)	−0.0028 (4)	−0.0028 (4)	−0.0014 (4)

### Geometric parameters (Å, °)

**Table:**

N1—C1	1.3509 (15)	O1—H1A	0.8200
N1—C2	1.3668 (15)	O2—N3	1.2302 (14)
N1—H1	0.8600	O3—N3	1.2348 (14)
N2—C1	1.3355 (15)	O4—N4	1.2404 (15)
N2—H2A	0.8600	O5—N4	1.2291 (15)
N2—H2B	0.8600	O6—C13	1.2331 (15)
C1—C5	1.4181 (16)	O7—C13	1.3067 (15)
C2—C3	1.3652 (17)	N3—C8	1.4546 (15)
C2—H2	0.9300	N4—C10	1.4563 (15)
C3—C4	1.4164 (18)	C7—C8	1.4208 (16)
C3—C6	1.5019 (18)	C7—C12	1.4372 (16)
C4—C5	1.3681 (18)	C8—C9	1.3861 (16)
C4—H4	0.9300	C9—C10	1.3794 (17)
C5—H5	0.9300	C9—H9	0.9300
C6—H6A	0.9600	C10—C11	1.3956 (17)
C6—H6B	0.9600	C11—C12	1.3793 (16)
C6—H6C	0.9600	C11—H11	0.9300
O1—C7	1.2957 (14)	C12—C13	1.4943 (16)
			
C1—N1—C2	123.48 (10)	O2—N3—O3	122.24 (11)
C1—N1—H1	118.2	O2—N3—C8	119.70 (10)
C2—N1—H1	118.3	O3—N3—C8	118.06 (11)
C1—N2—H2A	120.0	O5—N4—O4	123.31 (11)
C1—N2—H2B	120.0	O5—N4—C10	118.76 (11)
H2A—N2—H2B	120.0	O4—N4—C10	117.93 (11)
N2—C1—N1	119.16 (11)	O1—C7—C8	123.94 (11)
N2—C1—C5	123.65 (11)	O1—C7—C12	120.07 (10)
N1—C1—C5	117.18 (11)	C8—C7—C12	115.98 (10)
C3—C2—N1	121.14 (11)	C9—C8—C7	122.15 (11)
C3—C2—H2	119.4	C9—C8—N3	116.22 (10)
N1—C2—H2	119.4	C7—C8—N3	121.63 (10)
C2—C3—C4	116.59 (11)	C10—C9—C8	118.97 (11)
C2—C3—C6	122.06 (12)	C10—C9—H9	120.5
C4—C3—C6	121.34 (12)	C8—C9—H9	120.5
C5—C4—C3	122.13 (11)	C9—C10—C11	122.23 (11)
C5—C4—H4	118.9	C9—C10—N4	118.70 (11)
C3—C4—H4	118.9	C11—C10—N4	119.06 (11)
C4—C5—C1	119.43 (11)	C12—C11—C10	118.54 (11)
C4—C5—H5	120.3	C12—C11—H11	120.7
C1—C5—H5	120.3	C10—C11—H11	120.7
C3—C6—H6A	109.5	C11—C12—C7	122.12 (10)
C3—C6—H6B	109.5	C11—C12—C13	118.93 (10)
H6A—C6—H6B	109.5	C7—C12—C13	118.95 (10)
C3—C6—H6C	109.5	O6—C13—O7	123.31 (11)
H6A—C6—H6C	109.5	O6—C13—C12	120.35 (11)
H6B—C6—H6C	109.5	O7—C13—C12	116.34 (10)
C7—O1—H1A	109.5		
			
C2—N1—C1—N2	177.44 (11)	N3—C8—C9—C10	−178.15 (10)
C2—N1—C1—C5	−2.27 (17)	C8—C9—C10—C11	0.44 (18)
C1—N1—C2—C3	0.56 (19)	C8—C9—C10—N4	179.53 (11)
N1—C2—C3—C4	1.24 (18)	O5—N4—C10—C9	−175.21 (11)
N1—C2—C3—C6	−179.26 (12)	O4—N4—C10—C9	4.88 (17)
C2—C3—C4—C5	−1.29 (19)	O5—N4—C10—C11	3.91 (17)
C6—C3—C4—C5	179.21 (13)	O4—N4—C10—C11	−176.00 (11)
C3—C4—C5—C1	−0.4 (2)	C9—C10—C11—C12	−1.15 (18)
N2—C1—C5—C4	−177.56 (12)	N4—C10—C11—C12	179.76 (11)
N1—C1—C5—C4	2.14 (18)	C10—C11—C12—C7	0.51 (18)
O1—C7—C8—C9	179.70 (12)	C10—C11—C12—C13	179.74 (11)
C12—C7—C8—C9	−1.52 (17)	O1—C7—C12—C11	179.60 (11)
O1—C7—C8—N3	−1.24 (19)	C8—C7—C12—C11	0.77 (17)
C12—C7—C8—N3	177.54 (10)	O1—C7—C12—C13	0.37 (17)
O2—N3—C8—C9	−171.71 (11)	C8—C7—C12—C13	−178.46 (10)
O3—N3—C8—C9	8.51 (17)	C11—C12—C13—O6	−0.21 (18)
O2—N3—C8—C7	9.17 (18)	C7—C12—C13—O6	179.05 (11)
O3—N3—C8—C7	−170.60 (11)	C11—C12—C13—O7	−179.85 (11)
C7—C8—C9—C10	0.96 (18)	C7—C12—C13—O7	−0.59 (16)

### Hydrogen-bond geometry (Å, °)

**Table:**

*D*—H···*A*	*D*—H	H···*A*	*D*···*A*	*D*—H···*A*
O1—H1A···O7	0.82	1.66	2.4200 (13)	152
N1—H1···O6^i^	0.86	1.82	2.6768 (14)	174
N2—H2A···O7^i^	0.86	2.11	2.9668 (15)	176
N2—H2B···O1^ii^	0.86	2.16	2.8468 (15)	137
N2—H2B···O2^ii^	0.86	2.40	3.1723 (16)	149
C2—H2···O4^iii^	0.93	2.49	3.4073 (16)	169
C4—H4···O3^iv^	0.93	2.39	3.2371 (16)	151
C5—H5···O2^ii^	0.93	2.44	3.2328 (17)	143

Symmetry codes: (i) −*x*+1, −*y*+1, −*z*; (ii) *x*+1, *y*, *z*; (iii) *x*−1, *y*−1, *z*; (iv) −*x*+1, −*y*+1, −*z*+1.
